# The uncommon function and mechanism of the common enzyme glyceraldehyde-3-phosphate dehydrogenase in the metamorphosis of *Helicoverpa armigera*

**DOI:** 10.3389/fbioe.2022.1042867

**Published:** 2022-10-18

**Authors:** Wenli Zhao, Bo Zhang, Zichen Geng, Yanpeng Chang, Jizhen Wei, Shiheng An

**Affiliations:** State key Laboratory of Wheat and Maize Crop Science/Henan International Laboratory for Green Pest Control/College of Plant Protection, Henan Agricultural University, Zhengzhou, China

**Keywords:** GAPDH, 20E, metamorphosis, Treh1, glucose content

## Abstract

Glyceraldehyde-3-phosphate dehydrogenase (GAPDH), a key enzyme in glycolysis, is commonly used as an internal reference gene in humans, mice, and insects. However, the function of GAPDH in insect development, especially in metamorphosis, has not been reported. In the present study, *Helicoverpa armigera* and *Spodoptera frugiperda* ovarian cell lines (Sf9 cells) were used as materials to study the function and molecular mechanism of GAPDH in larval metamorphosis. The results showed that *HaGAPDH* was more closely related to GAPDH of *S. frugiperda* and *Spodoptera litura*. The transcript peaks of *HaGAPDH* in sixth instar larvae were 6L-3 (epidermal and midgut) and 6L-1 (fat body) days, and 20E and methoprene significantly upregulated the transcripts of *HaGAPDH* of larvae in qRT-PCR. HaGAPDH–GFP–His was specifically localized in mitochondria in Sf9 cells. Knockdown of *HaGAPDH* by RNA interference (RNAi) in sixth instar larvae resulted in weight loss, increased mortality, and decreases in the pupation rate and emergence rates. *HaGAPDH* is directly bound to soluble trehalase (HaTreh1) physically and under 20E treatment in yeast two-hybrid, coimmunoprecipitation, and colocalization experiments. In addition, knockdown of *HaGAPDH* increased the Treh1 activity, which in turn decreased the trehalose content but increased the glucose content in larvae. Therefore, these data demonstrated that GAPDH controlled the glucose content within the normal range to ensure glucose metabolism and metamorphosis by directly binding with HaTreh1.

## 1 Introduction

The best-known function of glyceraldehyde-3-phosphate dehydrogenase (GAPDH) is serving as the housekeeping gene. GAPDH is widely used as an internal reference in real-time PCR and Western blot (Chapman and Waldenstrom, 2015; Nie et al., 2017). In fact, GAPDH catalyzes glyceraldehyde-3-phosphate to 1,3-bisphosphoglycerate (1,3-BPG), the first reaction that consumes some energy in glucose after several steps without energy production in glycolysis (Yang et al., 2018). Therefore, the multifunctional enzyme GAPDH acts as the key enzyme in glycolysis and the center of glucose metabolism (Kornberg et al., 2018).

In humans, GAPDH has been shown to be involved in a variety of pathologies, such as diabetes (Yego and Mohr 2010) and several types of cancer (Zhou et al., 2008; Colell et al., 2009). For instance, Müller cells are a crucial component of the retinal tissue and maintain the blood–retinal barrier, and diabetes usually caused the loss of Müller cells. In the diabetic retina of rats, nuclear accumulation of GAPDH caused the loss of Müller cells (Kusner et al., 2004). GAPDH is also considered a new potential therapeutic target for cardiac disease states characterized by oxidative stress. Knockdown of GAPDH by siRNA in the H9C2 cardiomyoblasts decreased oxidant stress and cell apoptosis, enhanced autophagy in rotenone-induced H9C2 cells, increased antioxidant pathways, and preserved cell energy (Liang et al., 2015). Colony-stimulating factor 1 (CSF1), a macrophage cytokine, is strongly correlated with poor prognosis in ovarian cancer patients. Also, serum CSF-1 was used as a sensitive tumor marker, its elevated levels heralding disease recurrence or progression. In the human ovarian surface epithelial cell line, GAPDH stabilized the transcript and enhanced CSF1 protein levels by binding to CSF1 mRNA. The half-lives of CSF-1 mRNA were decreased by 50% in the presence of GAPDH siRNA (Zhou et al., 2008).

Research studies on GAPDH in insects are less than those of humans and mice. In insects, GAPDH is almost used as the reference gene. GAPDH was the development stage reference gene in *Rhopalosiphum padi* (Li et al., 2021), the tissue reference gene in *Lymantria dispar* (Yin et al., 2020), the reference gene in *Apolygus lucorum* for different sexes and entomopathogen infection studies (Luo et al., 2020), and the development profile study reference gene in *Tuta absoluta* (Yan et al., 2021). In *Helicoverpa armigera*, GAPDH was used as the reference gene for nuclear polyhedrosis viral infection (Zhang et al., 2015). In insects, GAPDH has other functions in addition to the reference gene. In *Laodelphax striatellus*, GAPDH is involved in resistance to autophagy induced by rice black-streaked dwarf virus (RBSDV) infection. RBSDV interacts with *LsGAPDH in vivo* and *in vitro*. Knockdown of *LsGAPDH* significantly reduces RBSDV infection-induced autophagy. RBSDV promotes the phosphorylation of AMPK and then leads to the phosphorylation and translocation of GAPDH from the cytoplasm to the nucleus, and phosphorylated GAPDH activates autophagy to inhibit RBSDV infection (Wang et al., 2022). Bisphenol S (BPS) is the industrial alternative to the endocrine disruptor bisphenol A (BPA).

Glycolysis starts with glucose (Zhong et al., 2018). In insects, trehalose is the blood sugar (Tatun N et al., 2008). The hydrolysis of trehalose to glucose, which is catalyzed by trehalase (Treh), is necessary for insect life activities. As the only known enzyme that irreversibly decomposes trehalose into glucose, Treh has two forms including soluble trehalase (soluble trehalase, Treh1) and membrane-bound trehalase (membrane-bound trehalase, Treh2), which are mainly classified by the transmembrane structure at the C-terminus (Shukla et al., 2015). Treh1 is crucial for insects. In the larvae midgut of *Omphisa fuscidentalis*, the Treh1 activity accounts for most of the total Treh activity, and 20-hydroxyecdysone (20E) increases Treh1 transcripts and the enzymatic activity, thus ensuring the normal development of the larvae (Tatun et al., 2008). In *Spodoptera exigua*, ds*Treh1*-injected larvae had a 50% mortality rate and molting failure (Chen et al., 2010). Knockdown of *NlTreh1* in *N. lugens* caused larval abnormities in molting and wings (Tang et al., 2017). In *Tribolium castaneum*, RNAi of Treh1a resulted in a mortality rate of up to 30% (Tang et al., 2016). In *Leptinotarsa decemlineata*, RNAi of Treh1a induced a mortality rate of up to 80% (Shi et al., 2016). In *H. armigera*, knockdown of Treh1 in the larvae led to weight loss and mortality increased. 20E enhanced the direct binding of HaTreh1 to HaATPs-α and controlled the production of ATP, thus affecting the growth and development of larvae (Chang et al., 2022).

In our previous study, *HaGAPDH* was identified as a HaTreh1-binding protein by a yeast two-hybrid (Y2H) screening library experiment (Chang et al., 2022). Considering the key roles of HaTreh1 in molting and metamorphosis of *H. armigera*, *HaGAPDH* in glycolysis, and HaTreh1 in glucose production, we proposed the following questions, what is the relationship between these two enzymes HaTreh1 and *HaGAPDH*? In addition to serving as an internal reference gene in *H. armigera* larvae, does GAPDH have other functions, and is it involved in larvae metamorphosis similar to HaTreh1? To answer the aforementioned scientific questions, quantitative real-time PCR (qRT-PCR) was performed to study the expression profile of *HaGAPDH*, RNA interference was used to explore the function of *HaGAPDH* in larval metamorphosis, and Y2H and coimmunoprecipitation (Co-IP) experiments were performed to prove the interaction between *HaGAPDH* and HaTreh1. We explored the function and mechanism of *HaGAPDH* in larval metamorphosis and provided experimental evidence and theoretical basis for targeting GAPDH for pest control in the future.

## 2 Materials and methods

### 2.1 Materials

#### 2.1.1 Insects

*Helicoverpa armigera* eggs were purchased from the Baiyun Industrial Company (Baiyun, Jiyuan, China) and reared in a laboratory using an artificial diet at 26°C, 75% humidity, and a 14L: 10D photoperiod (Zhao et al., 2005).

#### 2.1.2 Cell culture

The *Spodoptera frugiperda* ovarian cell line (Sf9 cells) was cultured at 28°C with the Sf-900 II serum-free medium, which contains 10% fetal bovine serum (Gibco) and 0.5% penicillin–streptomycin liquid (HyClone).

#### 2.1.3 Compound

Ecdysterone was purchased from the Solarbio Company (Solarbio, Beijing, China) and dissolved with DMSO. Methoprene was purchased from the MedChemExpress Company (MCE, Princeton, United States) and dissolved with DMSO.

## 3. Methods

### 3.1 Bioinformatic analysis

The open reading frame (ORF) of *HaGAPDH* (LOC110377691) was amplified by specific primers (Table 1) and sequenced by the Tsingke Biotechnology Company (Tsingke, Beijing, China). The phylogenetic tree was constructed by the neighbor-joining (NJ) method with MEGA 11.0 software.

**TABLE 1:** Primers used in the article.

Primer	Sequence (5'-3')	Usage
*HaGAPDH*-CDS-F	ATG​TCC​AAA​ATC​GGT​ATC​AAC	CDS clone
*HaGAPDH*-CDS-R	TTA​ATC​CTT​GGT​CTG​GAT​GTA	CDS clone
*HaGAPDH*-qPCR-F	AAG​CCC​GCT​ACT​TAC​GAT​GC	Specific expression analysis
*HaGAPDH*-qPCR-R	GTT​GGA​GTA​GCC​GAA​CTC​GT	Specific expression analysis
Ha18S-qPCR-F	GCA​TCT​TTC​AAA​TGT​CTG​C	Specific expression analysis
Ha18S-qPCR-R	TAC​TCA​TTC​CGA​TTA​CGA​G	Specific expression analysis
Ha-β-actin-qPCR-F	CCT​GGT​ATT​GCT​GAC​CGT​ATG​C	Specific expression analysis
Ha-β-actin-qPCR-R	CTG​TTG​GAA​GGT​GGA​GAG​GGA​A	Specific expression analysis
*HaGAPDH*-Ri-F	GCG​TAA​TAC​GAC​TCA​CTA​TAG​GGT​TCA​AGG​GCT​CCG​TTG​ACA​T	dsRNA synthesis
*HaGAPDH*-Ri-R	GCG​TAA​TAC​GAC​TCA​CTA​TAG​GGG​TTC​AGA​GCG​GGA​ATG​ACC​T	dsRNA synthesis
GFP-Ri-F	GAT​CAC​TAA​TAC​GAC​TCA​CTA​TAG​GGA​GAC​ACA​AGT​TCA​GCG​TGT​CCG	dsRNA synthesis
GFP-Ri-R	GAT​CAC​TAA​TAC​GAC​TCA​CTA​TAG​GGA​GAG​TTC​ACC​TTG​ATG​CCG​TTC	dsRNA synthesis
*HaGAPDH*-GFP-SacI-F	GCA​TCG​TTA​ACA​CGT​CAA​GAG​CTC ATG​TCC​AAA​ATC​GGT​ATC​AAC	Overexpression in Sf9 cells
*HaGAPDH*-GFP-Bgl Ⅱ-R	CTG​CAG​GCG​CGC​CGA​GAT​CTG​ATC​CTT​GGT​CTG​GAT​GTA	Overexpression in Sf9 cells
*HaGAPDH*-pGADT7-Sfi I-F	TAT​AGG​CCA​TTA​CGG​CCA​TGT​CCA​AAA​TCG​GTA​TCA​AC	Y2H
*HaGAPDH*-pGADT7-Sfi I-R	ATA​TGG​CCG​AGG​CGG​CCA​TCC​TTG​GTC​TGG​ATG​TA	Y2H

### 3.2 Quantitative real-time PCR (qRT-PCR)

The TRIzol method and HiScript III RT SuperMix for qPCR (+gDNA wiper) (R323–01, Vazyme) were used to get the total RNA and cDNA, respectively. Then, qRT-PCR was performed by using the ChamQ Universal SYBR qPCR Master Mix (Q711-02-03) (Vazyme) and a real-time qPCR instrument (Eppendorf). There were 10 μl 2 × ChamQ Universal SYBR qPCR Master Mix, 0.4 μl forward primer, 0.4 μl reverse primer, 1 μl cDNA template, and 8.2 μl ddH_2_O in the qRT-PCR system. The reaction program was as follows: 95°C (5 min), followed by 40 cycles of 95°C (15 s) and 60°C (20 s). Two reference genes *Ha18s* (AB620126.1, product size 230 bp, PCR efficiency: 0.97) and *Ha-β-Actin* (EU527017.1, product size 144 bp, PCR efficiency: 0.979) were selected to normalize the expression of *HaGAPDH*. The PCR efficiency of *HaGAPDH* primers is 1.07 (product size 204 bp). Then, three larvae were collected as a single sample, and three biological replicates and three technical replicates were performed for each experiment. The Student’s *t*-test or Tukey test was used to compare the significant differences.

### 3.3 Hormone treatment

The sixth instar larvae about 2 h after molting were selected, and there were six larvae in each group. The control group was injected with 5 μl DMSO (diluted to 1:10,000 with 1 × PBS) per larvae, and the experimental group was injected with 5 μl 20E (1.2 μg) or 5 μl methoprene (0.5 μg) per larvae. Then, the epidermis and fat body were collected at 0, 1, 3, and 6 h after hormone injection. The experiments were performed in three biological replicates.

### 3.4 Subcellular localization

#### 3.4.1 Subcellular localization of *HaGAPDH*

The ORF (without stop codon) sequence of *HaGAPDH* was ligated into the GFP-pIEx vector to construct *HaGAPDH*-GFP-pIEx using specific primers (Table 1). Then, the *HaGAPDH*-GFP-pIEx plasmid was transfected into the Sf9 cells using the FuGENE HD Transfection Reagent (4 μl of the Transfection Reagent per 1 μg plasmid) (Promega, E2311) referred to the previous description (Blochlinger and Diggelmann, 1984). The Sf9 cells transfected with a GFP-pIEx plasmid were used as control. After 24 h, the LSM710 laser confocal microscope (Zeiss) was used to observe the green fluorescence and take pictures.

#### 3.4.2 The mitochondrial localization

The Sf9 cells that transfected with the *HaGAPDH*-GFP-pIEx plasmid for 24 h were incubated with Mito-Tracker Red CMXRos (C1049B, Beyotime, 50 nM) at 37°C for 15 min. Then, fluorescence images were photographed using an LSM710 laser confocal microscope (Zeiss).

#### 3.4.3 Colocalization

The *HaGAPDH*-GFP-pIEx and HaTreh1-RFP-pIEx plasmids were co-transfected into Sf9 cells. About 48 h later, the green and red fluorescence were observed and photographed using an LSM710 laser confocal microscope (Zeiss).

### 3.5 RNA interference

The DNA templates for ds*HaGAPDH* (507 bp) and ds*GFP* (MN623123, 420 bp, the control) synthesis were amplified by PCR using specific primers containing the T7 promoter core sequence (Table 1). Subsequently, ds*RNA* was synthesized using the MEGAscript RNAi kit (Thermo Fisher Scientific), and the quality of ds*RNA* was detected using a BioPhotometer (Eppendorf) and by agarose electrophoresis. The sixth instar larvae about 2 h after molting were selected, and there were 30 larvae in each group. The control group was injected with 5 μg ds*GFP* per larvae, and the experimental group was injected with 5 μg ds*HaGAPDH*. After 24 h, the abovementioned injection was repeated one time. The larval weights were recorded at 0, 24, 48, 72, 96, and 144 h after ds*RNA* injection, and the mortality rate, pupation rate, and emergence rate were also recorded. These experiments were performed in three biological replicates. The Student’s *t*-test was used to compare the significant differences.

### 3.6 Yeast two-hybrid

The *HaGAPDH*-pGADT7 and HaTreh1-pGBKT7 plasmids were constructed using specific primers (Table 1). Then, *HaGAPDH*-pGADT7 and HaTreh1-pGBKT7 were co-transformed into the AH109 yeast competent cells, according to the methods described previously (Wang et al., 2020), and then, the yeast was spread on the SD-TL, SD-THL, SD-THLA, and SD-TLHA + x-α- gal plates. The aforementioned plates were cultured at 37°C for 3 days. The AH109 cells that co-transformed pGBKT7-P53 and pGADT7-LargeT were the positive control, and the AH109 cells that co-transformed pGBKT7-laminC and pGADT7-LargeT were the negative control.

### 3.7 Coimmunoprecipitation

The *HaGAPDH*-GFP-pIEx and HaTreh1-RFP-pIEx plasmids were co-transfected into Sf9 cells (4 μg each plasmid for 7.2 × 10^6^ cells). After 24 h, the cells were treated with 20E (1 μM) for 3 h. Then, the cells were collected on ice using the radio immunoprecipitation assay (RIPA) lysis buffer. A part of the solution was taken out as the input, and the remaining solution was used to purify GFP-tagged proteins by using the GFP-tag IP/Co-IP Kit (Bio LinkedIn). Finally, the protein samples were subjected to Western blot experiments.

### 3.8 Endogenous substance content measurement

The sixth instar larvae about 2 h after molting were selected, and there were six larvae in each group. The control group was injected with 5 μg ds*GFP* per head, and the experimental group was injected with 5 μg ds*HaGAPDH*, and then, the midguts were collected at 24 h after ds*RNA* injection. Then, the soluble trehalase activity, trehalose content, and glucose content were measured according to the instructions of the trehalase activity assay kit (A150-1-1, Nanjing Jiancheng), trehalose content assay kit (G0552W, Grace), and glucose content assay kit (G0504W, Grace), respectively. Three biological replicates were performed, and each biological replicate included three technical replicates. The Student’s *t*-test was used to compare the significant differences.

## 4 Results

### 4.1 Bioinformatic analysis of the *HaGAPDH* sequence

**FIGURE 1:**
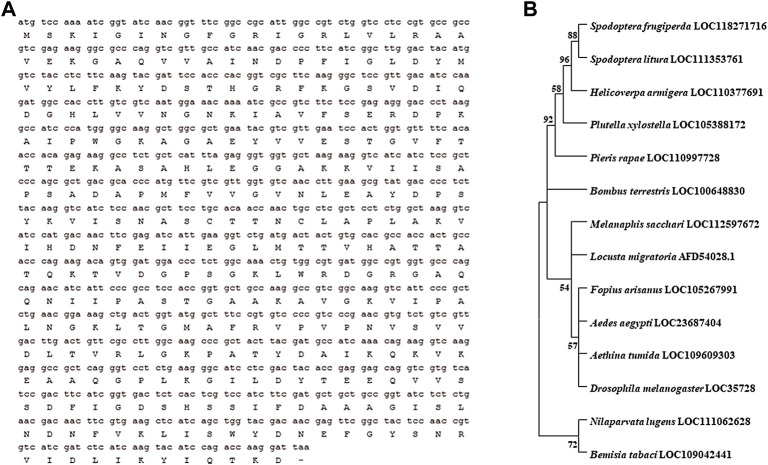
Bioinformatic analysis of the *HaGAPDH* sequence. **(A)** ORF sequence and the amino acid sequence of *HaGAPDH*. **(B)** Phylogenetic tree analysis of GAPDH. The tree was constructed by MEGA 11.0 software.

The length of *HaGAPDH* ORF is 525 bp and encodes 174 amino acid residues (Figure 1A). Phylogenetic analysis of *HaGAPDH* sequences in 30 species of insects, including Lepidoptera, Hemiptera, Diptera, and Coleoptera, showed that the *HaGAPDH* sequence of *H. armigera* was more closely related to those of *Spodoptera frugiperda* and *Spodoptera litura* (Figure 1B).

### 4.2 The expression profile of *HaGAPDH*

**FIGURE 2:**
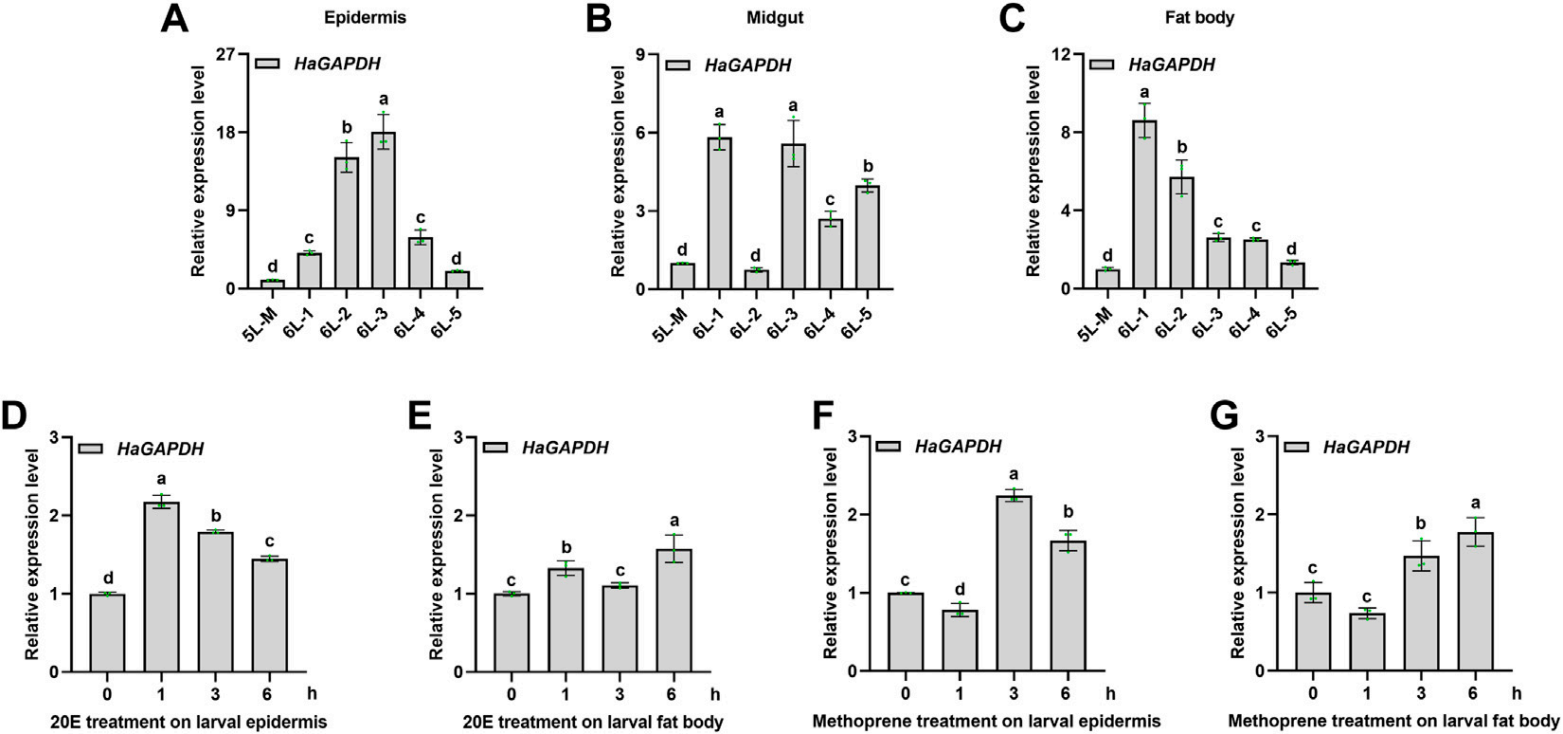
Expression profile of *HaGAPDH*. **(A–C)** Transcript analyses of *HaGAPDH* in the epidermis **(A)**, midgut **(B)**, and fat body **(C)** from 5L-M to 6L-5 days by qRT-PCR. 5L-M: the molting stage of the fifth instar larvae, 6L-1: the first day of the sixth instar larvae, **(D)** and **(E)** the transcript changes of *HaGAPDH* in the epidermis **(D)** and fat body **(E)** under the 20E treatment. Larvae were treated with 1.2 μg 20E (5 μl) or DMSO (5 μl) for 1, 3, and 6 h **(F)** and **(G)** the transcript changes of *HaGAPDH* in the epidermis **(F)** and fat body **(G)** under methoprene treatment. Larvae were treated with 0.5 μg methoprene (5 μl) or DMSO (5 μl) for 1, 3, and 6 h. *Ha18s* and *β-actin* were used as the reference genes. The error bars indicated the mean ± s.d. of three independent biological experiments and three technical repetitions. Different letters indicate significant differences at the *p* < 0.05 level using the Tukey test.

To study the function of *HaGAPDH* in larval development, its expression pattern was analyzed first. The results of qRT-PCR showed that *HaGAPDH* was expressed in all three tissues, namely, the epidermis, midgut, and fat body (Figures 2A–C). Also, during the period from the molting stage of the fifth instar (5L-M) to the fifth day of the sixth instar (6L-5), the transcript peaks of *HaGAPDH* were 6L-3 days in the epidermis (Figure 2A), 6L-1 and 6L-3 days in the midgut (Figure 2B), and 6L-1 day in the fat body (Figure 2C), respectively. Furthermore, the hormone treatment experimental data showed that both 20E and methoprene upregulated the transcript levels of *HaGAPDH* in the epidermis and fat body (Figures 2D–G).

### 4.3 *HaGAPDH* localized in mitochondria

**FIGURE 3:**
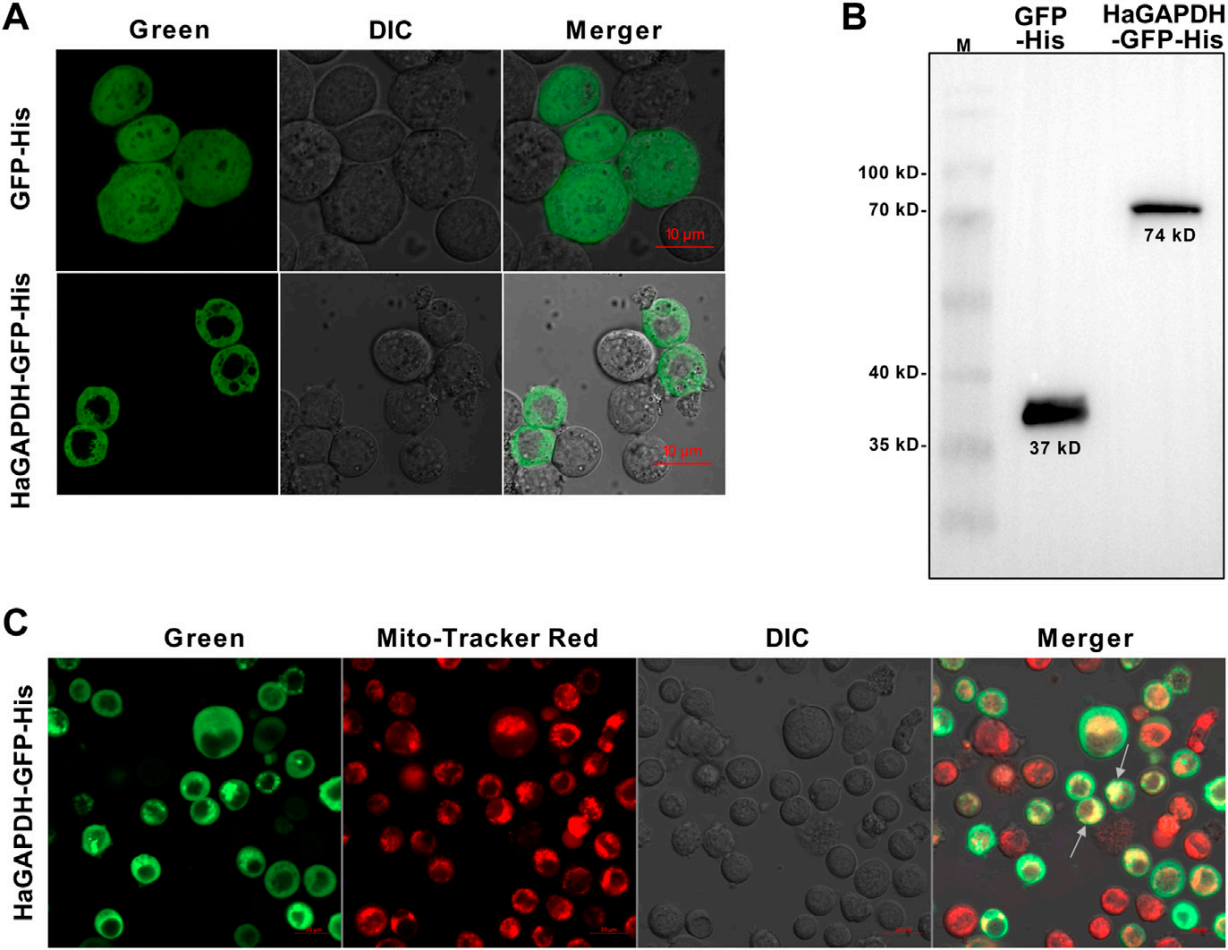
Mitochondrial localization of *HaGAPDH*. **(A)** Subcellular localization of *HaGAPDH*-GFP-His in Sf9 cells using an LSM710 confocal microscope. GFP-His was the control. The merger was the overlap of the green and bright fluorescence. **(B)** Expression of GFP-His (37 kD) and *HaGAPDH*-GFP-His (74 kD) in Sf9 cells by Western blot. M: protein marker. **(C)** *HaGAPDH*–GFP–His merged with mitochondria. The pictures were taken by using an LSM710 microscope. *HaGAPDH*–GFP–His overexpression cells were dyed with Mito-Tracker (50 nM) for 15 min. The merger was the overlap of the green fluorescence, Mito-Tracker red, and bright.

To explore the organelle in which *HaGAPDH* works, the subcellular localization of *HaGAPDH* in the Sf9 cells was studied. Compared to the GFP control that localized both in the cytoplasm and nucleus, the green fluorescence of the *HaGAPDH*-GFP protein was distributed in the cytoplasm (Figure 3A). The Western blot also proved the successful expression of GFP and *HaGAPDH*-GFP proteins in Sf9 cells (Figure 3B). Further mitochondrial staining experiments proved that the green fluorescence of the *HaGAPDH*-GFP protein specifically overlapped with the red fluorescence of mitochondria, which suggested the mitochondrial distribution of *HaGAPDH*-GFP (Figure 3C).

### 4.4 Knockdown of *HaGAPDH* caused the larval development defect

**FIGURE 4:**
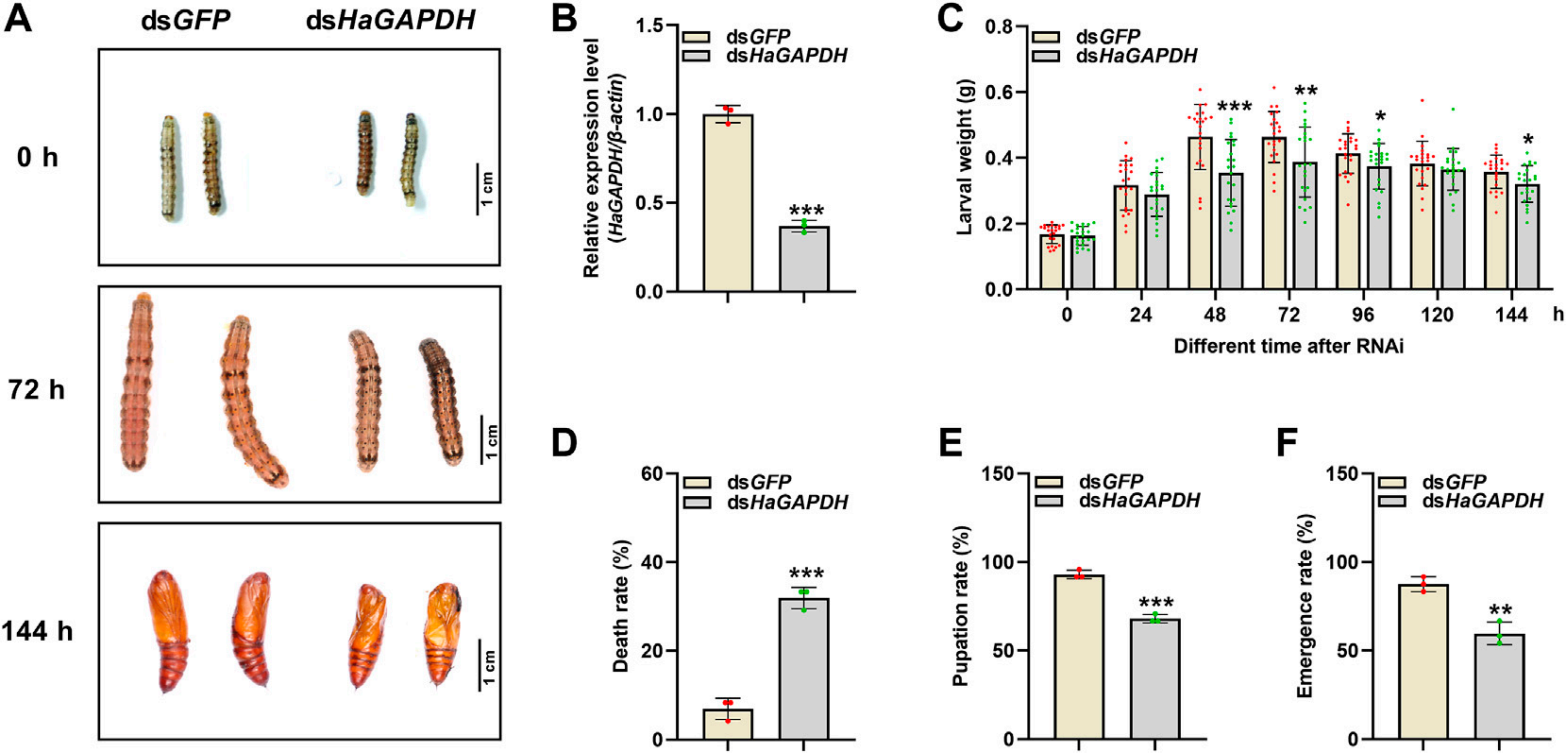
Effects on the sixth instar larvae after knockdown of *HaGAPDH* **(A)** and **(B)** the knockdown phenotypes **(A)** and efficiency **(B)** of *HaGAPDH* on the sixth instar larvae. The larvae were injected with 5 μg ds*HaGAPDH* or ds*GFP* (the control); then, photographs were taken at different timepoints, and midguts were collected for qRT-PCR at 24 h. *β-actin* was used as the reference gene. Error bars indicated the mean ± s.d. of three independent biological experiments and three technical repetitions. ***p < 0.001; Student’s *t-*test. **(C)** Larval weight changes after being injected with ds*RNA* for 24, 48, 72, 96, 120, and 144 h. There were 30 larvae in each group, and three independent biological experiments were performed. Each dot represented a repetition, and the error bars indicated mean ± SE. *p < 0.05, **p < 0.01, and ***p < 0.001; Student’s *t-*test. **(D–F)** Statistical analyses of the death rate **(D)**, pupation rate **(E)**, and emergence rate **(F)** after ds*RNA* injection. There were 30 larvae in each group. The error bars indicated the mean ± s.d. of three independent biological experiments. **p < 0.01 and ***p < 0.001; Student’s *t-*test.

RNAi was employed to specifically analyze the function of *HaGAPDH* in larval development. Compared with the ds*GFP* control, after the knockdown of *HaGAPDH*, the growth and development of ds*HaGAPDH-*injected larvae were abnormal (Figures 4A,B). In the ds*HaGAPDH*-injected group, the larval body weights were markedly reduced at 48, 72, 96, and 144 h after ds*RNA* injection (Figure 4C), and the final mortality rate was significantly increased (Figure 4D). In addition, the pupation rate and emergence rate of the experimental group were both significantly decreased (Figures 4E,F). The aforementioned data suggested the necessary role of *HaGAPDH* in larval development and metamorphosis.

### 4.5 *HaGAPDH* directly bound with HaTreh1

**FIGURE 5:**
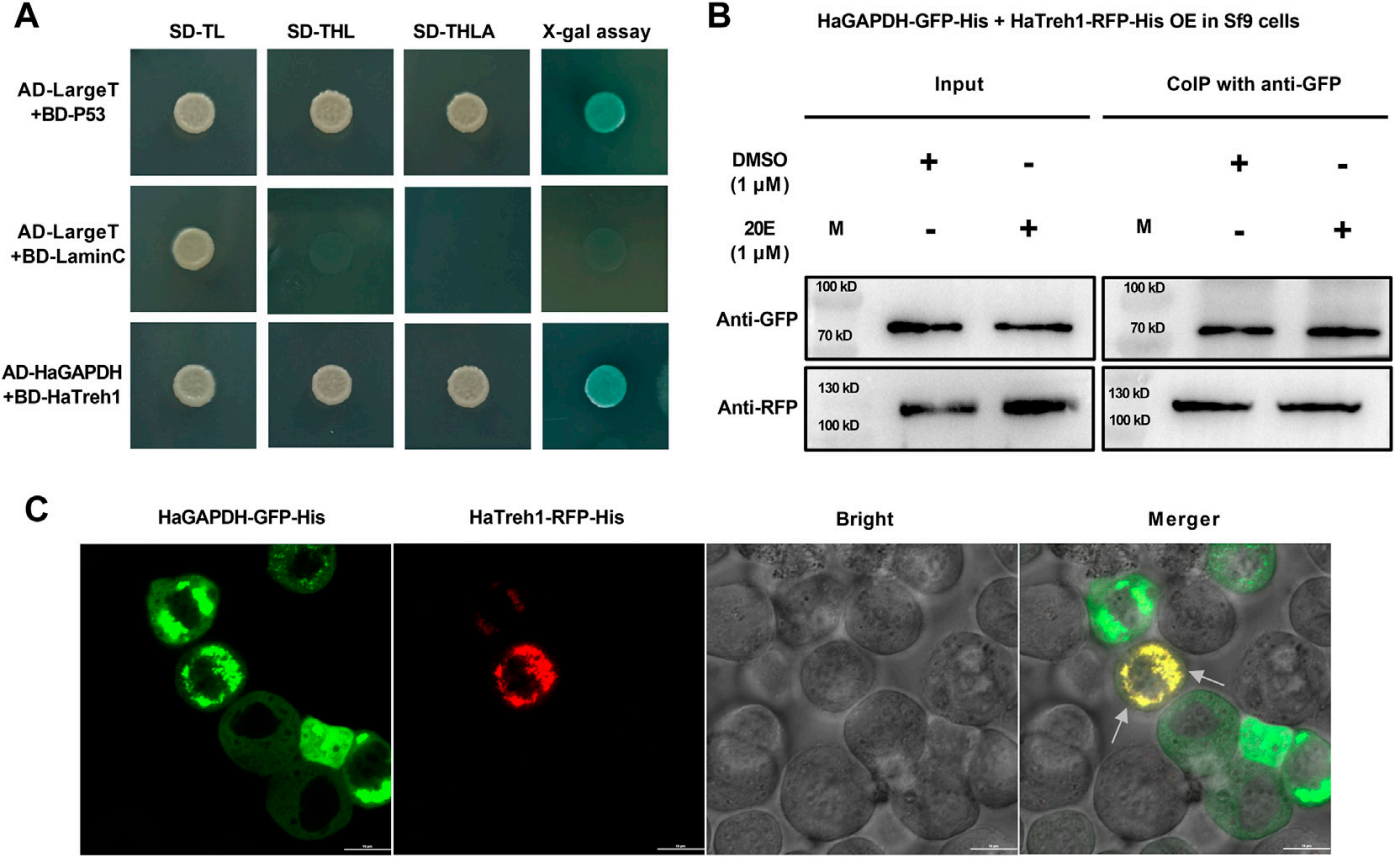
*HaGAPDH* bound to HaTreh1. **(A)** Y2H demonstrated the direct binding of BD-HaTreh1 and AD-*HaGAPDH*. AD-LargeT + BD-P53 was the positive control, and AD-LargeT + BD-laminC was the negative control. **(B)** Co-IP experiment proved the interaction between HaTreh1–RFP–His (103 kD) and *HaGAPDH*–GFP–His (74 kD) under the 20E treatment in Sf9 cells. M: protein marker. **(C)** Colocalization of HaTreh1–RFP–His and *HaGAPDH*–GFP–His in Sf9 cells by using the LSM710 microscope. The merger was the overlap of red and green fluorescence. The arrow indicates the overlapped yellow color.

To further analyze the functional mechanism of *HaGAPDH*, a series of experiments were performed to identify the binding protein of *HaGAPDH*. *HaGAPDH* was proven as the HaTreh1 binding protein in the previous Y2H library screening. Therefore, the Y2H point-to-point experiment was performed. Also, the results showed that the growth of yeast co-transfected with pGADT7-*HaGAPDH* and pGBKT7-HaTreh1 was consistent with that of the positive control pGADT7-LargeT + pGBKT7-P53 and was opposite to that of the negative control pGADT7-LargeT + pGBKT7-laminC (Figure 5A), indicating the direct binding of *HaGAPDH* and HaTreh1. The Co-IP experiment data proved the direct binding of *HaGAPDH* and HaTreh1 both in the control and under the 20E treatment (Figure 5B). The fluorescence photographs showed that the red fluorescence of HaTreh1-RFP overlapped the green fluorescence of *HaGAPDH*-GFP, thus showing yellow fluorescence (Figure 5C), which suggested the colocalization of *HaGAPDH* and HaTreh1. Accordingly, we inferred that *HaGAPDH* is directly bound with HaTreh1.

### 4.6 Knockdown of *HaGAPDH* changed the Treh1 activity and glucose content

**FIGURE 6:**
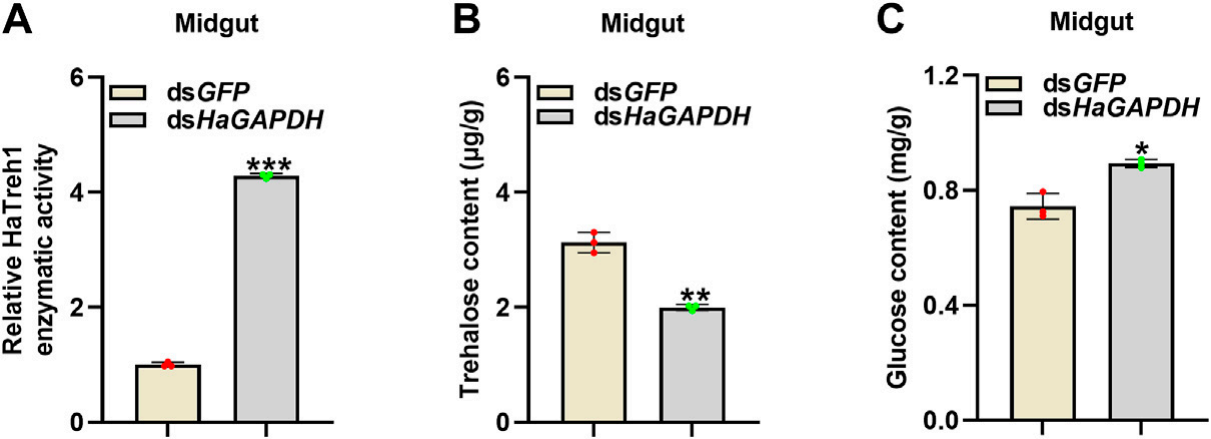
*HaGAPDH* controlled the Treh1 activity and sugar contents. **(A–C)** Treh1 activity **(A)**, trehalose content **(B)**, and glucose content **(C)** in the midguts after being injected with ds*GFP* (5 μg) or ds*HaGAPDH* (5 μg) for 24 h. Error bars indicated the mean ± s.d. of three independent biological experiments and three technical repetitions. *p < 0.05, **p < 0.01, and ***p < 0.001; Student’s *t-*test.

Considering the binding of *HaGAPDH* and HaTreh1, the corresponding endogenous sugar contents in larvae were measured. After the knockdown of *HaGAPDH* by RNAi, the enzymatic activity of HaTreh1 in the midgut was significantly increased by contrast with the ds*GFP* control (Figure 6A), and the trehalose content decreased but glucose increased correspondingly (Figures 6B,C).

## 5 Discussion

Glucose is the main source of energy for insect life. Therefore, GAPDH, a key enzyme in glycolysis, plays a crucial role in insects. In the present study, we proved that *HaGAPDH* plays an important role in maintaining the growth and promoting normal metamorphosis of *H. armigera* larvae. The transcript level of *HaGAPDH* was upregulated by 20E, and the knockdown of *HaGAPDH* by RNAi resulted in decreased larval weight, increased mortality, reduced pupation rate, and emergence rate. GAPDH controlled the Treh1 activity and glucose content of larvae by directly binding to HaTreh1. Our results revealed the critical function and molecular mechanism of GAPDH in larval metamorphosis.

### 5.1 Glyceraldehyde-3-phosphate dehydrogenase is crucial for larval development and metamorphosis

GAPDH has been considered the housekeeping gene in various species including humans, mice, and insects (Chapman and Waldenstrom, 2015; Nie et al., 2017). However, studies in humans have showed that the mRNA level of *GAPDH* in different tissues is different. The mRNA expression of *GAPDH* in the skeletal muscle was the highest in 72 human tissues, and *GAPDH* had a high expression trend in the high-energy-requirement tissues (Barber et al., 2005). In the present study, our results also proved that the transcript level peaks of *HaGAPDH* were attained in 6L-3 days in the larval epidermis and midgut, the period with high energy consumption (Figure 2). However, the transcripts of *HaGAPDH* in the fat body declined from 6L-1 and were the lowest from 6L-3 to 6L-5 days, with the pre-pupation period and huge energy demand (Figure 2). Since the fat body is the tissue that biosynthesizes trehalose and stores glycogen, the *HaGAPDH* expression pattern is reasonable for glycogen storage in the fat body. The transcript peaks of *HaGAPDH* in the epidermis and midgut appeared at 6L-3 (Figure 2) when the 20E titers increased in *H. armigera* larvae (Kang et al., 2019). The 20E treatment upregulated the transcripts of *HaGAPDH* (Figure 2), which is consistent with its expression peak at 6L-3 (Figure 2). It also suggested the potential role of *HaGAPDH* in larval metamorphosis. 20E is a steroid hormone (Dubrovsky, 2005), and our results are consistent with the fact that the steroid hormone estradiol increased *GAPDH* transcripts in the endometrium of ovariectomized ewes (Ing and Zhang, 2004). In addition, we also found that methoprene, an important hormone regulating larval growth and metamorphosis (Dubrovsky, 2005), also upregulated the transcripts of *HaGAPDH* (Figure 2). This dynamically regulated expression pattern of GAPDH is similar to the studies in other species (Jacob et al., 2013. In human ovarian cell lines HOSE6-3 and HOSE17-1, the *GAPDH* transcript is highly dynamic (Chao et al., 1990; Graven et al., 1994; Polati et al., 2012; Jacob et al., 2013). Northern blot results in monolayers of human WI-38/va13 cells, several cancer cells, and monkey cos-1 cells revealed that *GAPDH* transcripts were upregulated by the calcium ionophore A23187 (Chao et al., 1990). The *GAPDH* mRNA levels were increased under hypoxia treatment in ovine endothelial cells (Graven et al., 1994). In mouse bone marrow macrophages, GAPDH expression was upregulated by ferric ammonium citrate (Polati et al., 2012).

More importantly, further RNAi results proved the key role of *HaGAPDH* in larval development and metamorphosis (Figure 4). The larval phenotypes in the ds*HaGAPDH*-injected larvae were similar to those of important genes in the 20E pathway, such as the ecdysone receptor (EcR). Decreased EcR transcripts by RNAi in *Apolygus lucorum*, *Leptinotarsa decemlineata*, *Nilaparvata lugens*, and *Tribolium castaneum* led to molting defects and larval death (Tan and Palli, 2008; Wu et al., 2012; Tan et al., 2015; Xu et al., 2020). The transcript peaks, the upregulated transcript level by 20E, the larval metamorphosis defects after RNAi, and the previously mentioned results all fully demonstrated that *HaGAPDH* indeed plays an important role in larval metamorphosis. Our study is the first report on the function of GAPDH in insect metamorphosis.

### 5.2 Glyceraldehyde-3-phosphate dehydrogenase controls the glucose content in larvae by directly binding with HaTreh1

In the present study, our data proved that *HaGAPDH* is the mitochondria protein (Figure 3). This is the first evidence on the mitochondrial localization of GAPDH in insects. There are lots of reports about the sublocalization of GAPDH in human and mouse cells. For instance, the non-tetrameric forms of GAPDH localized in the nucleus of HeLa cells (Arutyunova et al., 2003). In HEK293 cells, GAPDH accumulated in the nucleus during apoptosis (Hara et al., 2005). In rat insulinoma INS-1 cells, GAPDH primarily showed cytoplasm localization (Park et al., 2009). The mitochondria-localized *HaGAPDH* was similar to those of PC12 cells and mice. Rotenone induces PC12 cell apoptosis and the translocation to mitochondria of GAPDH (Huang et al., 2009). GAPDH targets the mitochondria in the mouse heart (Kohr et al., 2014).

For the first time, *HaGAPDH* was proven as the HaTreh1 binding protein, and this binding also exists under 20E treatment (Figure 3). HaTreh1, a key enzyme for trehalose hydrolysis, directly binds to HaATPs-α which controls ATP production (Chang et al., 2022). *HaGAPDH* is another protein that directly binds to HaTreh1 in the present study. In a previous study, HaTreh1 has been reported to be working in the mitochondria (Chang et al., 2022). Also, the combination between *HaGAPDH* and HaTreh1 was consistent with the mitochondrial localization of *HaGAPDH*. There have been many studies on the binding protein of GAPDH. In HEK293 cells, GAPDH bounds to Sirtuin 1 (SIRT1), a nicotinamide adenine dinucleotide (NAD^+^)-dependent protein deacetylase (Chang et al., 2022). GAPDH was demonstrated as tubulin-binding protein (Tisdale et al., 2009; Landino et al., 2014). Similarly, GAPDH is also bound to actin (Waingeh et al., 2004). In mouse cell lines, GAPDH interacts with lactoferrin (Chauhan et al., 2015).

Interestingly, the knockdown of *HaGAPDH* increased the HaTreh1 activity and glucose content but decreased the trehalose content in larvae (Figure 6). Accordingly*,* we inferred that the activity of HaTreh1 was inhibited after interaction with *HaGAPDH*. The aforementioned data were reasonable for the phenotypes of ds*HaGAPDH*-injected larvae (Figure 4). After knockdown of the key enzyme in glycolysis *HaGAPDH*, the interaction between GAPDH and Treh1 led to an increase in the enzymatic activity of Treh1, thus hydrolyzing more trehalose into glucose; however, the ds*HaGAPDH*-injected larvae cannot use these sugar sources; finally, the imbalance of glucose metabolism led to the death and pupation failure of larvae. From this, we inferred that during the normal growth of larvae, GAPDH ensures the glucose content is at a normal level by interacting with Treh1 and controlling its enzymatic activity. This regulation model can prevent larvae from being unable to complete the process of sugar decomposition and energy production for too much glucose and ensure the development of larvae.

## Data Availability

The original contributions presented in the study are included in the article/Supplementary Material; further inquiries can be directed to the corresponding author.

## References

[B1] Arutyunova E. I. Danshina P. V. Domnina L. V. Pleten A. P. Muronetz V. I. (2003). Oxidation of glyceraldehyde-3-phosphate dehydrogenase enhances its binding to nucleic acids. Biochem. Biophys. Res. Commun. 307, 547–552. 10.1016/s0006-291x(03)01222-1 12893257

[B2] Barber R. D. Harmer D. W. Coleman R. A. Clark B. J. (2005). GAPDH as a housekeeping gene: Analysis of GAPDH mRNA expression in a panel of 72 human tissues. Physiol. Genomics 21, 389–395. 10.1152/physiolgenomics.00025.2005 15769908

[B3] Blochlinger K. Diggelmann H. (1984). Hygromycin B phosphotransferase as a selectable marker for DNA transfer experiments with higher eucaryotic cells. Mol. Cell. Biol. 4, 2929–2931. 10.1128/mcb.4.12.2929-2931.1984 6098829 PMC369308

[B4] Chang Y. Zhang B. Du M. Geng Z. Wei J. Guan R. (2022). The vital hormone 20-hydroxyecdysone controls ATP production by upregulating binding of trehalase 1 with ATP synthase subunit α in Helicoverpa armigera. J. Biol. Chem. 298, 101565. 10.1016/j.jbc.2022.101565 34999119 PMC8819028

[B5] Chao C. C. Yam W. C. Lin-Chao S. (1990). Coordinated induction of two unrelated glucose-regulated protein genes by a calcium ionophore: Human BiP/GRP78 and GAPDH. Biochem. Biophys. Res. Commun. 171, 431–438. 10.1016/0006-291x(90)91411-k 2118350

[B6] Chapman J. R. Waldenström J. (2015). With reference to reference genes: A systematic review of endogenous controls in gene expression studies. PLoS One 10, e0141853. 10.1371/journal.pone.0141853 26555275 PMC4640531

[B7] Chauhan A. S. Rawat P. Malhotra H. Sheokand N. Kumar M. Patidar A. (2015). Secreted multifunctional Glyceraldehyde-3-phosphate dehydrogenase sequesters lactoferrin and iron into cells via a non-canonical pathway. Sci. Rep. 5, 18465. 10.1038/srep18465 26672975 PMC4682080

[B8] Chen J. Tang B. Chen H. Yao Q. Huang X. Chen J. (2010). Different functions of the insect soluble and membrane-bound trehalase genes in chitin biosynthesis revealed by RNA interference. PLoS One 5, e10133. 10.1371/journal.pone.0010133 20405036 PMC2853572

[B9] Colell A. Green D. R. Ricci J. E. (2009). Novel roles for GAPDH in cell death and carcinogenesis. Cell Death Differ. 16 (12), 1573–1581. 10.1038/cdd.2009.137 19779498

[B10] Dubrovsky E. B. (2005). Hormonal cross talk in insect development. Trends Endocrinol. Metab. 16 (1), 6–11. 10.1016/j.tem.2004.11.003 15620543

[B11] Graven K. K. Troxler R. F. Kornfeld H. Panchenko M. V. Farber H. W. (1994). Regulation of endothelial cell glyceraldehyde-3-phosphate dehydrogenase expression by hypoxia. J. Biol. Chem. 269 (39), 24446–24453. 10.1016/s0021-9258(19)51104-8 7929107

[B12] Hara M. R. Agrawal N. Kim S. F. Cascio M. B. Fujimuro M. Ozeki Y. (2005). S-nitrosylated GAPDH initiates apoptotic cell death by nuclear translocation following Siah1 binding. Nat. Cell Biol. 7, 665–674. 10.1038/ncb1268 15951807

[B13] Huang J. Hao L. Xiong N. Cao X. Liang Z. Sun S. (2009). Involvement of glyceraldehyde-3-phosphate dehydrogenase in rotenone-induced cell apoptosis: Relevance to protein misfolding and aggregation. Brain Res. 1279, 1–8. 10.1016/j.brainres.2009.05.011 19445904

[B14] Ing N. H. Zhang Y. (2004). Cell-specific expression of estrogen-responsive genes in the uteri of cyclic, early pregnant and ovariectomized ewes. Theriogenology 62, 403–414. 10.1016/j.theriogenology.2003.10.017 15225997

[B15] Jacob F. Guertler R. Naim S. Nixdorf S. Fedier A. Hacker N. F. (2013). Careful selection of reference genes is required for reliable performance of RT-qPCR in human normal and cancer cell lines. PLoS One 8, e59180. 10.1371/journal.pone.0059180 23554992 PMC3598660

[B16] Kang X. L. Zhang J. Y. Wang D. Zhao Y. M. Han X. L. Wang J. X. (2019). The steroid hormone 20-hydroxyecdysone binds to dopamine receptor to repress lepidopteran insect feeding and promote pupation. PLoS Genet. 15, e1008331. 10.1371/journal.pgen.1008331 31412019 PMC6693746

[B17] Kohr M. J. Murphy E. Steenbergen C. (2014). Glyceraldehyde-3-phosphate dehydrogenase acts as a mitochondrial trans-S-nitrosylase in the heart. PLoS One 9, e111448. 10.1371/journal.pone.0111448 25347796 PMC4210263

[B18] Kornberg M. D. Bhargava P. Kim P. M. Putluri V. Snowman A. M. Putluri N. (2018). Dimethyl fumarate targets GAPDH and aerobic glycolysis to modulate immunity. Science 360, 449–453. 10.1126/science.aan4665 29599194 PMC5924419

[B19] Kusner L. L. Sarthy V. P. Mohr S. Domb A. J. Frucht-Pery J. (2004). Nuclear translocation of glyceraldehyde-3-phosphate dehydrogenase: A role in high glucose-induced apoptosis in retinal müller cells. Invest. Ophthalmol. Vis. Sci. 45 (5), 2543–1561. 10.1167/iovs.03-1294 15111614

[B20] Landino L. M. Hagedorn T. D. Kennett K. L. (2014). Evidence for thiol/disulfide exchange reactions between tubulin and glyceraldehyde-3-phosphate dehydrogenase. Cytoskelet. Hob. 71, 707–718. 10.1002/cm.21204 25545749

[B21] Li M. Li X. Wang C. Li Q. Zhu S. Zhang Y. (2021). Selection and validation of reference genes for qRT-PCR analysis of Rhopalosiphum padi (Hemiptera: Aphididae). Front. Physiol. 12, 663338. 10.3389/fphys.2021.663338 33935809 PMC8079785

[B22] Liang S. Figtree G. Aiqun M. Ping Z. (2015). GAPDH-knockdown reduce rotenone-induced H9C2 cells death via autophagy and anti-oxidative stress pathway. Toxicol. Lett. 234, 162–171. 10.1016/j.toxlet.2015.02.017 25725130

[B23] Luo J. Wang A. Cheng Y. Rong H. Guo L. Peng Y. (2020). Selection and validation of suitable reference genes for RT-qPCR analysis in Apolygus lucorum (Hemiptera: Miridae). J. Econ. Entomol. 113, 451–460. 10.1093/jee/toz301 31773146

[B24] Nie X. Li C. Hu S. Xue F. Kang Y. J. Zhang W. (2017). An appropriate loading control for Western blot analysis in animal models of myocardial ischemic infarction. Biochem. Biophys. Rep. 12, 108–113. 10.1016/j.bbrep.2017.09.001 28955798 PMC5613232

[B25] Park J. Han D. Kim K. Kang Y. Kim Y. (2009). O-GlcNAcylation disrupts glyceraldehyde-3-phosphate dehydrogenase homo-tetramer formation and mediates its nuclear translocation. Biochimica Biophysica Acta - Proteins Proteomics 1794, 254–262. 10.1016/j.bbapap.2008.10.003 19022411

[B26] Polati R. Castagna A. Bossi A. M. Alberio T. De Domenico I. Kaplan J. (2012). Murine macrophages response to iron. J. Proteomics 76, 10–27. 10.1016/j.jprot.2012.07.018 22835775

[B27] Shi J. F. Xu Q. Y. Sun Q. K. Meng Q. W. Mu L. L. Guo W. C. (2016). Physiological roles of trehalose in Leptinotarsa larvae revealed by RNA interference of trehalose-6-phosphate synthase and trehalase genes. Insect biochem. Mol. Biol. 77, 52–68. 10.1016/j.ibmb.2016.07.012 27524277

[B28] Shukla E. Thorat L. J. Nath B. B. Gaikwad S. M. (2015). Insect trehalase: Physiological significance and potential applications. Glycobiology 25, 357–367. 10.1093/glycob/cwu125 25429048

[B29] Tan A. Palli S. R. (2008). Edysone receptor isoforms play distinct roles in controlling molting and metamorphosis in the red flour beetle, *Tribolium castaneum* . Mol. Cell Endocrinol. 291, 42–49. 10.1016/j.mce.2008.05.006 18583027 PMC2595142

[B30] Tan Y. A. Xiao L. B. Zhao J. Sun Y. Bai L. X. (2015). Molecular and functional characterization of the ecdysone receptor isoform-A from the cotton mirid bug, Apolygus lucorum (Meyer-Dür). Gene 574, 88–94. 10.1016/j.gene.2015.07.085 26238700

[B31] Tang B. Wei P. Zhao L. Shi Z. Shen Q. Yang M. (2016). Knockdown of five trehalase genes using RNA interference regulates the gene expression of the chitin biosynthesis pathway in *Tribolium castaneum* . BMC Biotechnol. 16, 67. 10.1186/s12896-016-0297-2 27596613 PMC5011928

[B32] Tang B. Yang M. Shen Q. Xu Y. Wang H. Wang S. (2017). Suppressing the activity of trehalase with validamycin disrupts the trehalose and chitin biosynthesis pathways in the rice Brown planthopper, Nilaparvata lugens. Pestic. Biochem. Physiol. 137, 81–90. 10.1016/j.pestbp.2016.10.003 28364808

[B33] Tatun N. Singtripop T. Sakurai S. (2008). Dual control of midgut trehalase activity by 20-hydroxyecdysone and an inhibitory factor in the bamboo borer Omphisa fuscidentalis Hampson. J. Insect Physiol. 54, 351–357. 10.1016/j.jinsphys.2007.10.006 18023454

[B34] Tisdale E. J. Azizi F. Artalejo C. R. (2009). Rab2 utilizes glyceraldehyde-3-phosphate dehydrogenase and protein kinase cι to associate with microtubules and to recruit dynein. J. Biol. Chem. 284, 5876–5884. 10.1074/jbc.M807756200 19106097 PMC2645835

[B35] Waingeh V. F. Lowe S. L. Thomasson K. A. (2004). Brownian dynamics of interactions between glyceraldehyde-3-phosphate dehydrogenase (GAPDH) mutants and F-actin. Biopolymers 73, 533–541. 10.1002/bip.10560 15048777

[B36] Wang K. Li M. Q. Chang Y. P. Zhang B. Zhao Q. Z. Zhao W. L. (2020). The basic helix-loop-helix transcription factor OsBLR1 regulates leaf angle in rice via brassinosteroid signalling. Plant Mol. Biol. 102, 589–602. 10.1007/s11103-020-00965-5 32026326

[B37] Wang Q. Lu L. Zeng M. Wang D. Zhang T. Z. Xie Y. (2022). Rice black-streaked dwarf virus P10 promotes phosphorylation of GAPDH (glyceraldehyde-3-phosphate dehydrogenase) to induce autophagy in Laodelphax striatellus. Autophagy 18, 745–764. 10.1080/15548627.2021.1954773 34313529 PMC9037447

[B38] Wu W. J. Wang Y. Huang H. J. Bao Y. Y. Zhang C. X. (2012). Ecdysone receptor controls wing morphogenesis and melanization during rice planthopper metamorphosis. J. Insect Physiol. 58 (3), 420–426. 10.1016/j.jinsphys.2012.01.012 22326762

[B39] Xu Q. Y. Deng P. Zhang Q. Li A. Fu K. Y. Guo W. C. (2020). Ecdysone receptor isoforms play distinct roles in larval-pupal-adult transition in *Leptinotarsa decemlineata* . Insect Sci. 27, 487–499. 10.1111/1744-7917.12662 30688001 PMC7277042

[B40] Yan X. Zhang Y. Xu K. Wang Y. Yang W. (2021). Selection and validation of reference genes for gene expression analysis in Tuta absoluta meyrick (Lepidoptera: Gelechiidae). Insects 12, 589. 10.3390/insects12070589 34209609 PMC8305163

[B41] Yang J. S. Hsu J. W. Park S. Y. Li J. Oldham W. M. Beznoussenko G. V. (2018). GAPDH inhibits intracellular pathways during starvation for cellular energy homeostasis. Nature 561, 263–267. 10.1038/s41586-018-0475-6 30209366 PMC6152935

[B42] Yego E. C. Mohr S. (2010). siah-1 Protein is necessary for high glucose-induced glyceraldehyde-3-phosphate dehydrogenase nuclear accumulation and cell death in Muller cells. J. Biol. Chem. 285, 3181–3190. 10.1074/jbc.M109.083907 19940145 PMC2823464

[B43] Yin J. Sun L. Zhang Q. Cao C. (2020). Screening and evaluation of the stability of expression of reference genes in *Lymantria dispar* (Lepidoptera: Erebidae) using qRT-PCR. Gene 749, 144712. 10.1016/j.gene.2020.144712 32360412

[B44] Zhang S. An S. Li Z. Wu F. Yang Q. Liu Y. (2015). Identification and validation of reference genes for normalization of gene expression analysis using qRT-PCR in Helicoverpa armigera (Lepidoptera: Noctuidae). Gene 555, 393–402. 10.1016/j.gene.2014.11.038 25447918

[B45] Zhao X. F. An X. M. Wang J. X. Dong D. J. Du X. J. Sueda S. (2005). Expression of the Helicoverpa cathepsin B-like proteinase during embryonic development. Arch. Insect Biochem. Physiol. 58, 39–46. 10.1002/arch.20030 15599933

[B46] Zhong X. Y. Yuan X. M. Xu Y. Y. Yin M. Yan W. W. Zou S. W. (2018). CARM1 methylates GAPDH to regulate glucose metabolism and is suppressed in liver cancer. Cell Rep. 24, 3207–3223. 10.1016/j.celrep.2018.08.066 30232003

[B47] Zhou Y. Yi X. Stoffer J. B. Bonafe N. Gilmore-Hebert M. McAlpine J. (2008). The multifunctional protein glyceraldehyde-3-phosphate dehydrogenase is both regulated and controls colony-stimulating factor-1 messenger RNA stability in ovarian cancer. Mol. Cancer Res. 6, 1375–1384. 10.1158/1541-7786.MCR-07-2170 18708368 PMC2587019

